# Build-a-Cell: Engineering a Synthetic Cell Community

**DOI:** 10.3390/life11111176

**Published:** 2021-11-03

**Authors:** Caroline Frischmon, Carlise Sorenson, Michael Winikoff, Katarzyna P. Adamala

**Affiliations:** 1 Science Communication Lab., BioTechnology Institute, University of Minnesota, Minneapolis, MN 55108, USA; frisc109@umn.edu (C.F.); michaelw@umn.edu (M.W.); 2 Department of Genetics, Cell Biology and Development, University of Minnesota, Minneapolis, MN 55455, USA; soren776@umn.edu

**Keywords:** synthetic cells, artificial life, cell-free, liposomes, bioengineering, synthetic biology

## Abstract

Build-a-Cell is a global network of researchers that aims to develop synthetic living cells within the next decade. These cells will revolutionize the biotechnology industry by providing scientists and engineers with a more complete understanding of biology. Researchers can already replicate many cellular functions individually, but combining them into a single cell remains a significant challenge. This integration step will require the type of large-scale collaboration made possible by Build-a-Cell’s open, collective structure. Beyond the lab, Build-a-Cell addresses policy issues and biosecurity concerns associated with synthetic cells. The following review discusses Build-a-Cell’s history, function, and goals.

## 1. Introduction

Cells are nature’s simplest factories, taking in raw materials and transforming them into the chemicals and energy required for life. If we learned to program biological cells the way we design processing plants, one might envision a world of “pocket factories” that manufacture food and personalized medicine at home. It may sound like science fiction, but synthetic biologists envision this possibility if we can first achieve one of modern science’s most audacious goals—to create life in the form of a lab-made cell.

In 2017, a group of researchers committed to developing synthetic living cells estimated that reaching the finish line would take at least 5000 expert years of work [1]. If a lab of 10 worked for 500 years straight, it had a chance at creating synthetic life. However, these numbers obscure the highly interdisciplinary nature of the work required to build life—from DNA and protein synthesis to transport and energy control. No single lab has the expertise to achieve this feat on its own. Thus, an international network called Build-a-Cell formed to bridge the gap in expertise and resources. Seventy-two labs from 13 countries have joined the network so far. Experts now predict the finish line for developing the first synthetic cells within the decade. Here we explore the history and goals of the Build-a-Cell project, and the future of synthetic cells envisioned by Build-a-Cell researchers.

## 2. Synthetic Cell Overview

“What I cannot create, I do not understand.”—Richard Feynman

Our current inability to create cells prevents us from gaining a full understanding of life. These knowledge gaps necessitate a tinker-and-test approach to modifying living systems. We make a change in a DNA sequence and then check to see if it is useful without fully understanding the underlying mechanism. Many in the Build-a-Cell community see synthetic cell research as an opportunity to revolutionize this iterative approach to biological engineering [2].

Building a cell from scratch will create an operational understanding of life’s component parts and solve the remaining biological unknowns. That knowledge could offer our tinker-and-test bioindustry a more targeted, engineered strategy. Build-a-Cell expects to develop tools to program cells the way we program computers to accomplish tasks like designing new medicines or producing food in arid regions.

Build-a-Cell researchers agree on a general vision for building synthetic cells, but they diverge on the methodologies for getting there. Some take a top-down approach by starting with a living cell and simplifying its genome until it no longer resembles its natural state. This approach is simpler than its counterpart because it starts with a system already well-tuned for life. Those who work from the bottom up use chemical building blocks to engineer cells from scratch [3,4]. The end product of bottom-up synthesis may not even resemble what we currently think of as a cell. Airplanes, for example, fly but they do not look like birds. Approaching cell synthesis without constraining the biological definition could lead to similarly novel solutions. Regardless of what the cells might look like in the end, however, Build-a-Cell researchers are all committed to revolutionizing our current understanding of biotechnology.

While Build-a-Cell is the first synthetic cell organization that seeks to work across all geographical boundaries, several local other synthetic cell communities came together before Build-a-Cell to coordinate research efforts and raise funds [5]. Europe is leading the way with three organizations: the Dutch BaSyC consortium and the UK-based fabriCELL [4] are currently active, while the German Max Planck Research Network in Synthetic Biology (MaxSynBio) ended operations after a very successful tenure [6]. On the other side of the world, the Japanese Society for Cell Synthesis Research supports efforts of synthetic cell community in Japan [7].

## 3. Build-a-Cell Community Structure

**Figure 1:**
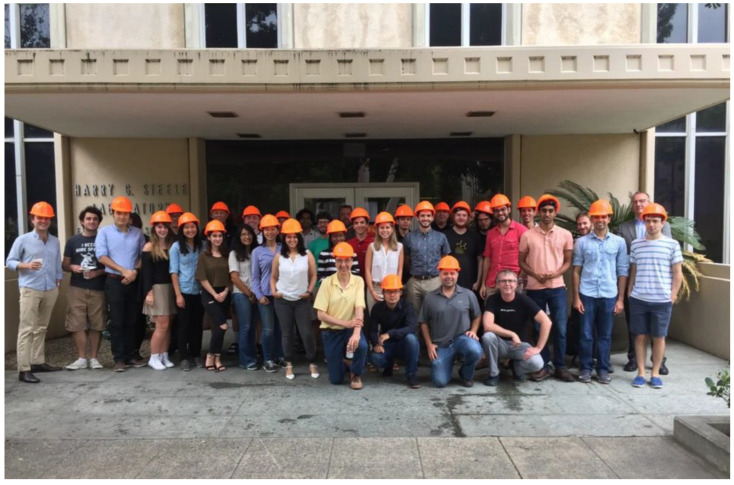
Build-a-Cell Hard Hat Workshop, California Institute of Technology, 24 July 2017, Pasadena, California. CC BY 2.0.

Build-a-Cell kicked off in 2017 with a hard hat workshop at the California Institute of Technology (Figure 1). The hard hat metaphor alluded to the nature of research in the new Build-a-Cell community. Participants were not simply engaged in fundamental research, they were on the path to constructing living cells.

At the hard hat meeting, Build-a-Cell took a unique approach to sparking collaboration by jettisoning the formal structure of a scientific conference. Instead of attending talks, participants joined interactive working groups to tackle roadblocks in synthetic cell research—from fundamental challenges to biosafety and biosecurity concerns. The working groups, which continue to meet biannually, provide an opportunity to devise new experiments, exchange protocols, and share research advances. Build-a-Cell has since hosted five in-person workshops and continued to meet virtually during the COVID-19 pandemic.

The working groups support a larger effort to establish a high-trust, collaborative environment among researchers. Build-a-Cell founders envision a culture similar to the internet’s open-source developer community, which created some of the world’s most innovative technologies through a collective effort on a global scale. Build-a-Cell, which hopes to create other life-changing technologies, models the internet’s open-source culture through Slack channels, working groups, and GitHub collaborations available to other researchers and the public.

The community also emphasizes sharing research findings broadly rather than hiding them behind paywalls and patents. As a community guideline, any information obtained using shared protocols or reagents must be publicly available online, even in the form of prepublished data. Kate Adamala, Build-a-Cell’s lead coordinator, supports an open-source approach but recognizes that it must be balanced with the need to produce valuable intellectual property (IP) that brings in private investment. “That’s how fields move forward”, she says. “Not just thorough academic research, but through entrepreneurship”. So far, only one company, Boston start-up Synlife, works specifically on engineering biomedical applications of synthetic cell technologies. Adamala hopes more will follow soon, as Build-a-Cell continues to grapple with optimizing research accessibility and private investment.

Even as they seek a balance, it’s clear the collective structure has ongoing advantages for Build-a-Cell. A significant roadblock in synthetic cell research is reproducibility among labs. Without standardized protocols, each research group must create its own experimental procedures by trial and error. To overcome that challenge and streamline collaboration, Build-a-Cell prioritized the development of shared research protocols.

They first tackled liposome synthesis by developing and publishing a protocol on GitHub that is easily followed, even by new graduate students. This early success further encouraged a culture of shared standardized protocols. A team at the National Institute of Standards and Technology (NIST) led by Elizabeth Strychalski, a member of the Build-a-Cell steering committee, now works specifically on developing protocols for synthetic cells and cell-free systems. Standardized research techniques make experiments more transferable between labs, so the pace of research between Build-a-Cell labs picks up as more protocols are established.

## 4. Remaining Challenges in Synthetic Cell Development

Build-a-Cell researchers can already program most cellular functions individually [8], from genome replication [9,10,11] to ATP generation [12,13,14,15,16]. As these successes pile up, the next challenge for the community is to combine functions and “boot up” a fully functioning cell [17]. “If you just put the parts together at equilibrium, it’s likely you’ve made a dead cell”, explains John Glass, a Build-a-Cell co-founder, “because one of the characteristics of life are these disequilibria, where you have movement of metabolites in both directions across membranes.” Booting up the cell will require labs to integrate all specialized processes at disequilibrium in a single, living cell.

Integration will be a tremendous task since advancements in one cellular process are not easily combined with another. Furthermore, most labs are highly specialized, working with specific biological subsystems, and lack experience putting all the puzzle pieces together.

Interlab, one of the working groups formed at Build-a-Cell’s first workshop, began addressing the incompatibilities between synthetic cell subsystems. Labs within the working group send their components to others that then integrate those components into their own cell systems. From there, researchers determine which ones work and which need further modification prior to integration. For example, Interlab members exchange parts for engineering robust, multicomponent genetic circuits in bacterial cell-free translation systems.

Interlab had just established an effective workflow for the component exchange when the COVID-19 pandemic interrupted their plans. Researchers were barred from lab spaces, and upon returning, were constrained by limited schedules and social distancing requirements. The restrictions slowed the pace of experimentation, but Interlab found other ways to collaborate by hosting monthly virtual meetings and participating in online forums, both of which are publicly available at buildacell.org (accessed on 15 September 2021).

## 5. Build-a-Cell Goals Outside the Lab

Build-a-Cell researchers in the U.S. are discussing the promise of synthetic cells with policymakers at the federal level with the goal of establishing a national science policy on synthetic cell technology. They want the government to designate synthetic cell development as a priority because of the promise it holds for public health and national security. Priority designation would create funding opportunities and attract talented engineers to the field, a necessity if Build-a-Cell is to maintain its global leadership in science and technology.

Drew Endy, Build-a-Cell co-founder, is a member of the White House working group on synthetic biology. The group will establish a roadmap for synthetic biology that informs how the country should handle the monumental changes synthetic cells will deliver. Endy offers four pillars that guide his concept for a roadmap.

### 5.1. Mastering Cell Synthesis

The first pillar, mastering cell synthesis, will provide a more complete, operational understanding of biology. By eliminating our remaining biological blind spots, this work will redirect the way we currently approach biotechnology. For example, the ability to engineer synthetic cell metabolism will aid in our understanding of natural metabolism and allow us to build better bioproduction strains.

### 5.2. Building the Bionet

The next pillar, building the bionet, is an important step toward implementing synthetic cell technology. The bionet, still more than a decade away, describes networked biotechnology where DNA sequences will be shared as digital information on the internet. The bionet will allow engineers to design organisms in labs and deploy them anywhere in the world as quickly as information is passed along on the internet. Life-saving DNA sequences for food and medicine may become downloadable like any other internet file [18].

### 5.3. Securing the Bionet

Although the bionet provides powerful benefits, it is vulnerable to many of the same cybersecurity threats the internet continues to face. Therefore, securing the bionet stands as the third pillar in Endy’s roadmap to ensure protections against hackers and those with nefarious intent. Endy hopes to learn from the mistakes of the internet’s early days in forming a more robust plan for securing the bionet.

### 5.4. Democratizing the Bioeconomy

Finally, the fourth pillar reflects the role the public should play in the synthetic cell industry. Endy believes as citizens of the bioeconomy, all people should have access to the tools necessary to read and write DNA, just as anyone can engage with the internet. Democratizing networks, whether the internet or bionet, empowers individuals to solve problems with information available at their fingertips. The democratization of the bioeconomy, along with the other three pillars of his plan, guides Endy’s vision for a domestic synthetic cell policy that safely uplifts all citizens.

Build-a-Cell’s policy focus extends to international funding strategies as well. Current international collaborations must be funded separately at the domestic level because research grants are limited to a national scale. This system is especially taxing for smaller countries with fewer funding opportunities. Build-a-Cell works closely with its European counterpart, Synthetic Cell EU, to overcome this logistical challenge. Together they are lobbying the European Commission, and the National Science Foundation in the United States, to set up multinational initiatives that fund projects connecting both sides of the pond.

While researchers from all over the globe may join Build-a-Cell, seed organizations outside the U.S. encourage participants to focus on regionally specific initiatives involving synthetic cells. Build-a-Cell South America recently formed from a partnership between Build-a-Cell researchers in the U.S. and the National Institute of Science and Technology in Brazil. The new group draws attention to the vital role of synthetic cell research in protecting biodiversity in regions like the Amazon. Elibio Rech, the founder of Build-a-Cell South America, explains that the ability to reproduce unique traits in synthetic organisms will allow us to leverage biodiversity without destroying it. While still mainly based in Brazil, the daughter organization hopes to engage and activate researchers throughout South America in the global effort toward synthetic cell synthesis.

## 6. Public Concerns Regarding Synthetic Cells

When the first synthetic cell comes to life, it will be the only organism on Earth not shaped by the checks and balances imposed by evolution over the last 4 billion years. This raises some biosafety concerns that Build-a-Cell researchers need to address long before they have established robust living cells. Like the debate surrounding genetically modified organisms (GMOs), there is concern over what may happen if the synthetic cells escape containment and enter natural ecosystems. To address this, engineers can encode a safety switch into the DNA of synthetic cells that kills them off while leaving the surrounding natural microbiome untouched.

There are also biosafety and biosecurity implications of the technology, including the potential for bad actors to intentionally or accidentally misuse aspects of synthetic cell technologies. Build-a-Cell researchers understand these concerns, as similar conversations have followed other scientific advancements. Focusing only on negative applications, however, eclipses the important benefits of new technologies. Even without synthetic cells, readily available biotechnology makes biological attacks with viruses and other organisms possible. Synthetic cell research could provide tools that combat the threats we already face by equipping bioengineers with a more complete understanding of biology.

The COVID-19 pandemic exemplifies another threat exacerbated by our incomplete understanding of life. Imagine a world where, three days after discovering COVID-19, a vaccine was available in every pharmacy in the world. Such a future may be possible if we understand biology so intimately that we no longer require a year’s worth of research and testing to develop a new vaccine. The ability to grow synthetic cells onsite at pharmacies could also eliminate the massive logistical challenges in vaccine distribution. Now more than ever, we must confront the threat posed by not developing synthetic cells despite the ethical and biosafety concerns.

For better or for worse, the major societal impacts of synthetic cells remain far off. Along the way, however, conversations with bioethicists, health and safety advisors, and other experts will inform the technology’s future. A current challenge, for example, involves the classification of synthetic biological materials, which do not fit into the standard Biological Safety Level (BSL) categorization. A top-down organism may be classified in the category of the parent organism, but the bottom-up approach used by many Build-a-Cell engineers requires a new framework and new safety precautions. This is an ongoing conversation within the Build-a-Cell community.

## 7. Conclusions

Synthetic cells hold transformative potential for our society. The operational understanding of life resulting from their synthesis will provide important insights into current and future biological vulnerabilities, including disease and bioterrorism. A more complete understanding of cells will also move the bioeconomy past its tinker-and-test approach toward a deliberate strategy designed to rationally implement drug development and bioproducts manufacturing solutions.

Engineering synthetic life is one of our generation’s greatest scientific challenges. Researchers can only succeed through the widespread collaboration made possible by communities like Build-a-Cell. The network does more than cultivate collaboration among researchers, however. It democratizes the development of synthetic cells through its emphasis on open-access research and inclusive participation. Anyone may join the Build-a-Cell community and engage in ongoing discussions through online channels.

In many ways, the scale of collaboration and accessibility in Build-a-Cell echoes the culture, as well as the dangers that surrounded the development of the internet. Contributors from all over the world created a tool that is available at nearly everyone’s fingertips, rather than concentrated in the hands of the powerful few. Build-a-Cell hopes to create the same outcome for synthetic cells: a life-changing technology that reaches everyone and reshapes biological engineering along the way.
